# Home Ultrasound: A Contemporary and Valuable Tool for Palliative Medicine

**DOI:** 10.7759/cureus.55573

**Published:** 2024-03-05

**Authors:** Ana Carolina Monteiro, Tomás França de Santana, Mariana Morais, Catarina Santos, João Aurélio, Inês Santos, Sofia Cruz, Dolores Vázquez, Sheila Ferreira Arroja, José Mariz

**Affiliations:** 1 Internal Medicine Department, Hospital Prof. Doutor Fernando Fonseca, Amadora, PRT; 2 Radiology Department, Hospital CUF Tejo, Lisboa, PRT; 3 Internal Medicine Department, Centro Hospitalar Lisboa Central - Hospital São José, Lisboa, PRT; 4 Internal Medicine Department, Hospital Garcia de Orta, Almada, PRT; 5 Internal Medicine Department, Centro Hospitalar do Algarve - Unidade Hospitalar de Portimão, Portimão, PRT; 6 Internal Medicine Department, Centro Hospitalar Lisboa Ocidental - Hospital Egas Moniz, Lisboa, PRT; 7 Internal Medicine Department, Hospital Vila Franca de Xira, Vila Franca de Xira, PRT; 8 Emergency Department, Hospital de Braga, Braga, PRT; 9 Life and Health Sciences Research Institute (ICVS), School of Medicine, University of Minho, Braga, PRT

**Keywords:** hospice care, home-based palliative care, handheld ultrasound, pocus, point-of-care ultrasound, palliative care, home ultrasound

## Abstract

This narrative review explores the application of point-of-care ultrasound (POCUS) in palliative care and its feasibility in home care settings. POCUS has the potential to streamline diagnostic strategies without patient transfer to the hospital, expedite timely symptomatic relief, and reduce complications from specific palliative interventions. The advent of handheld ultrasound devices has made it an attractive diagnostic and interventional adjunct in acute palliative care. POCUS has gained widespread acceptance as part of routine care in emergency medicine and intensive care, guiding certain procedures and increasing their safety. The modernization and miniaturization of ultrasound equipment have made ultra-portable devices available, allowing for better-quality images at affordable prices. Handheld devices have the potential to revolutionize everyday clinical practice in home-based palliative care, contributing to important bedside clinical decisions.

Palliative care patients often require diagnostic examinations in the last months of their lives, with CT being the most frequently performed imaging procedure. However, CT imaging is associated with high costs and burdens, leading to increased suffering and impaired quality of life. Clinical ultrasound, a dialogic imaging modality, offers a safer and more efficient approach to palliative care. POCUS applications, which are cost-effective, non-invasive, and well-tolerated, can be used to improve patient satisfaction and diagnostic understanding.

POCUS is a valuable tool in palliative care, improving diagnostic accuracy and reducing the time to diagnosis for various pathologies. It is a standard of care for many procedures and improves patient safety. However, there are limitations to POCUS in palliative care, such as operator-dependent examination variability and limited availability of trained professionals. To overcome these limitations, palliative care physicians should receive mandatory training in POCUS, which can be incorporated into the core curriculum. Additionally, ultrasound teleconsulting can assist less experienced examiners in real-time examinations. The literature on POCUS in palliative care is limited, but research on patient-oriented outcomes is crucial. POCUS should be considered a supplement to good clinical reasoning and regulated radiological evaluations.

## Introduction and background

Palliative care is a comprehensive approach to the care of patients suffering from severe, life-threatening illnesses and their families. It aims to enhance their quality of life by attending to their medical and psychological symptoms, addressing their social and spiritual needs, and mitigating their distress [1].

The combination of a progressively older population and enhanced survival rates in both malignant and non-malignant medical conditions has resulted in a growing cohort of populations who experience advanced or end-stage chronic illnesses. While cancer continues to be the most commonly diagnosed condition among individuals receiving palliative care, patients with advanced non-malignant diseases, such as cardiovascular and chronic respiratory disorders, experience similar levels of symptom burden and require equal levels of care [2].

Patients requiring palliative care may present themselves in acute care settings, such as internal medicine wards or emergency rooms, due to acute decompensation, unsuccessful implementation of planned management regimens, or disease progression [3,4]. Medical assessment can occur in several venues, including hospital services, outpatient medical clinics, hospices, and even through home visits. The decisions made during these interactions are crucial, as they have the potential to significantly impact the course of patient care [5].

Palliative care is a paradigm change from a focus on disease treatment and management to the provision of comfort and preservation of dignity for patients, with the aim of enhancing their overall quality of life. Hence, it is imperative to implement palliative care at an early stage in the progression of any illness that imposes limitations on life expectancy. This approach ensures timely alleviation of symptoms and proper management of intercurrent complications [6].

The incorporation of imaging techniques within contemporary palliative care practices can offer supplementary advantages. However, it is crucial to approach this integration with caution, taking into account the delicate equilibrium between the potential benefits and risks involved. It is imperative to avoid engaging in futile care when the potential benefits of imaging are outweighed by its potential harm [7].

Point-of-care ultrasound (POCUS) has been defined as the performance of ultrasound imaging by the treating clinician at the patient’s bedside to provide immediate answers to specific clinical questions, generally dichotomous, and guide clinical care [8]. It refers to the use of ultrasound as an extension of the traditional physical examination for clinical decision-making in scenarios where ultrasound is cost-effective (high sensitivity and negative predictive value) [9]. Research has demonstrated that engaging in this practice enhances one’s proficiency in conducting comprehensive physical examinations and augments diagnostic precision, hence enhancing the quality of clinical reasoning [10]. Its purpose is not to replace a regular examination performed by a radiologist or cardiologist [11].

The utilization of POCUS has garnered extensive recognition as a standard component of healthcare in the fields of emergency medicine and intensive care. Its application extends beyond diagnostic goals, encompassing the facilitation of specific procedures and enhancing their safety [12]. The performance attributes of POCUS render it a compelling diagnostic and interventional tool in the context of acute palliative care.

The advancements in modernizing and reducing the size of ultrasound equipment have facilitated the availability of ultra-portable devices, which enable the acquisition of high-quality images at cost-effective rates. Certain ultrasonic scanners are equipped with a solitary multi-frequency probe, enabling the selection of the most appropriate configuration for the intended scan [13]. They have storage capacity, which enables the documentation of clinically relevant pathological findings in the patient’s medical record as POCUS examinations are undertaken as part of the clinical examination.

Furthermore, it is worth noting that there is also the possibility of real-time upload of images to a server, thereby allowing the examiner to evaluate the observations, juxtapose them with prior examinations, and dynamically assess a patient’s clinical state. This process can be facilitated by a remote specialist who can conduct a remote analysis and provide valuable feedback [14]. Artificial intelligence plays a crucial role in the optimization of pictures and the safe guidance of invasive procedures [15].

Several validation studies have been performed to evaluate the feasibility and accuracy of handheld POCUS devices in comparison to high-end ultrasound systems. These studies have consistently shown a high level of diagnostic agreement between the two types of devices [16].

The availability of handheld ultrasound machines has expanded the potential applications of POCUS beyond inpatient settings, making point-of-care imaging site-independent and available in many non-hospital environments, including home visits, hospice care, and community nursing facilities [17].

Hence, portable electronic devices can significantly transform routine clinical procedures within the context of home-based palliative care, thereby facilitating the formulation of critical clinical judgments at the patient’s bedside.

This narrative review aims to enhance comprehension regarding the effectiveness of POCUS in palliative care and the viability of portable ultrasound in the context of home care. It examines the utilization of POCUS in a range of diagnostic and therapeutic interventions within the field of palliative care medicine. It aims to shed light on the advantages, constraints, and difficulties associated with POCUS, while also advocating for the integration of handheld ultrasound devices in ambulatory palliative care settings. The article explores the potential of POCUS to optimize diagnostic approaches, eliminating the need for patient transport to the hospital. Additionally, it highlights how POCUS might enhance the prompt alleviation of symptoms and minimize complications from specific palliative interventions.

## Review

Methodology

A database search was performed utilizing the Medical Subject Headings (MESH) terms “palliative medicine,” “palliative care,” “home care services,” “home care system,” “hospice care,” “point-of-care ultrasound,” “point-of-care systems,” “point-of-care diagnostic imaging,” “ultrasound,” and “ultrasonography.”

A comprehensive search of academic databases, including PubMed-Medline, PubMed Central, Scopus, Cochrane, Embase, and Scielo, was conducted. Filters were used to include open-access journals currently published in the field of medicine. The scope of the search was restricted to studies published in 2000 or subsequent years. The existing body of literature in the field of palliative care units is limited, consisting primarily of a few reviews and descriptive studies in palliative care units. As a result of the progress made in POCUS over the past quarter-century, it was anticipated that no pertinent research would have been undertaken before 2000. There were no restrictions imposed on the language of publishing for the search.

The comprehensive investigation yielded a compilation of 156 significant scholarly articles. Upon conducting a thorough examination, we chose a total of 130 items that satisfactorily fulfilled the predetermined criteria and evoked the necessary real curiosity for our research.

POCUS in home-based palliative care

Several studies have documented a rise in the number of diagnostic examinations conducted during the latter months of a patient’s life, with CT becoming the most commonly utilized imaging modality [18]. CT imaging is frequently employed in clinical practice to elucidate acute symptoms, assess disease progression, and make informed decisions regarding the continuation or modification of treatment protocols [7]. However, it is important to note that this particular issue is linked to substantial financial expenses and frequently imposes considerable hardship on individuals receiving palliative care. This is primarily due to the need for transportation to healthcare facilities, extended waiting periods within emergency departments, heightened levels of anxiety, and emotional distress. Ultimately, these factors exacerbate the already existing suffering and diminish the overall quality of life for patients who require compassionate care, reassurance, and empathy, rather than an emphasis on further diagnostic procedures and unattainable prospects of recovery [19].

Assessing the impact of intensive imaging approaches on patient outcomes has posed challenges, and it is frequently observed that treatment modifications based on imaging findings may not yield favorable outcomes for patients nearing the end of their lives. Hence, it is imperative to thoroughly evaluate the advantages and drawbacks of comprehensive imaging during the terminal stage of life, taking into account factors such as comorbidities, the patient’s quality of life, and their personal preferences. Adopting a rational and patient-centered approach is crucial in making informed decisions regarding the potential benefits and therapeutic implications of such imaging procedures [20].

In contrast to CT, clinical ultrasonography is characterized as a “dialogic” imaging technique that facilitates direct contact between doctors and patients. It can be seamlessly incorporated into the clinical examination process and employed immediately at the patient’s bedside, eliminating any temporal or spatial barriers. The patient’s perception of undivided attention is enhanced by the security derived from having the examination conducted by a practitioner who is both known and trusted by the patient, as well as by the utilization of dialogue and ultrasound-specific skin contact [21]. Therefore, it is not unexpected that individuals in need of palliative care highly appreciate the presence and skill of doctors utilizing POCUS at their bedside. This leads to a boost in patient satisfaction and a shared comprehension of the diagnostic process [22].

Palliative care professionals frequently depend exclusively on the information obtained from the patient’s medical history and physical examination during home visits, as the time-sensitive nature of their patients’ diseases necessitates prompt medical decision-making. The utilization of handheld devices as a diagnostic tool in patients receiving palliative care enables the symptom-oriented acquisition of reliable imaging information, enhancing the range of diagnostic and therapeutic options available for managing symptom aggravation at the patient’s bedside. By mitigating uncertainty and minimizing diagnostic delays, this process facilitates prompt alleviation of symptoms and, due to its repeatability, enables subsequent care and valuable insights into treatment efficacy [23]. Handheld devices provide the potential to serve as an interventional tool, as they have the capability to efficiently guide procedures that are specifically targeted, thereby mitigating complications and contributing to the enhancement of patient safety [24]. Ultrasound examinations conducted in hospice settings or during home care have the potential to offer valuable insights that might aid in prognostic talks and facilitate collaborative decision-making.

POCUS applications offer several advantages compared to traditional radiological imaging methods. POCUS is a cost-effective tool that does not require invasive procedures and is well tolerated by patients. Additionally, POCUS avoids the use of ionizing radiation, which can be harmful, and reduces the risk of renal damage caused by intravenous contrast in debilitated patients. This is particularly important as renal failure in this population can lead to increased morbidity [25]. Given the limited availability of physicians for home visits, particularly in rural areas, providing non-physician healthcare providers with the necessary training in performing POCUS can be an option to enhance outpatient palliative care [26].

The portability of portable devices holds significant importance in the context of palliative care, as a majority of patients in this stage of illness express a desire to spend their final phase of life in the comfort of their own homes, contingent upon the availability of appropriate treatment [27].

The utilization of this technological assistance facilitates the identification of intercurrent conditions or the performance of invasive procedures, thereby minimizing the necessity, in the majority of instances, to transfer patients to a hospital setting, which can potentially impose physical and emotional burdens on both patients and caregivers. Ultrasound-based home care effectively mitigates the occurrence of adverse symptoms, such as pain, nausea, or exhaustion, by minimizing unneeded emergency room visits, transfers, and repositioning necessitated for other imaging examinations. Consequently, this approach not only enhances symptom management but also enhances the overall quality of life for patients [28]. Ultimately, the primary objective of POCUS in palliative care is to empower technology reaching out to the community, rather than the terminally ill patient reaching out to seek hospital admission [29].

Research findings have indicated that the utilization of handheld devices in ambulatory settings has played a significant role in around 50% of treatment decisions. These decisions encompass many aspects such as adjustments in drug regimens and the implementation of therapeutic interventions. This observation underscores the noteworthy influence of handheld devices on the management of patients [26].

Additionally, the utilization of POCUS assessment has the potential to assist in the prioritization of patients for admission to an emergency medicine unit. It can even serve as the initial step in the diagnostic evaluation process for patients in an outpatient setting who do not have a pre-existing malignancy. This holds greater significance for patients residing in rural and isolated areas, as they encounter obstacles in accessing fair healthcare, including limitations in travel and financial burdens associated with standard diagnostic imaging services [30]. One potential strategy for mitigating these clinical issues involves the implementation of portable and readily available bedside ultrasound machines, which can be operated by certified healthcare professionals.

Applications of POCUS in home-based palliative care

**Figure 1:**
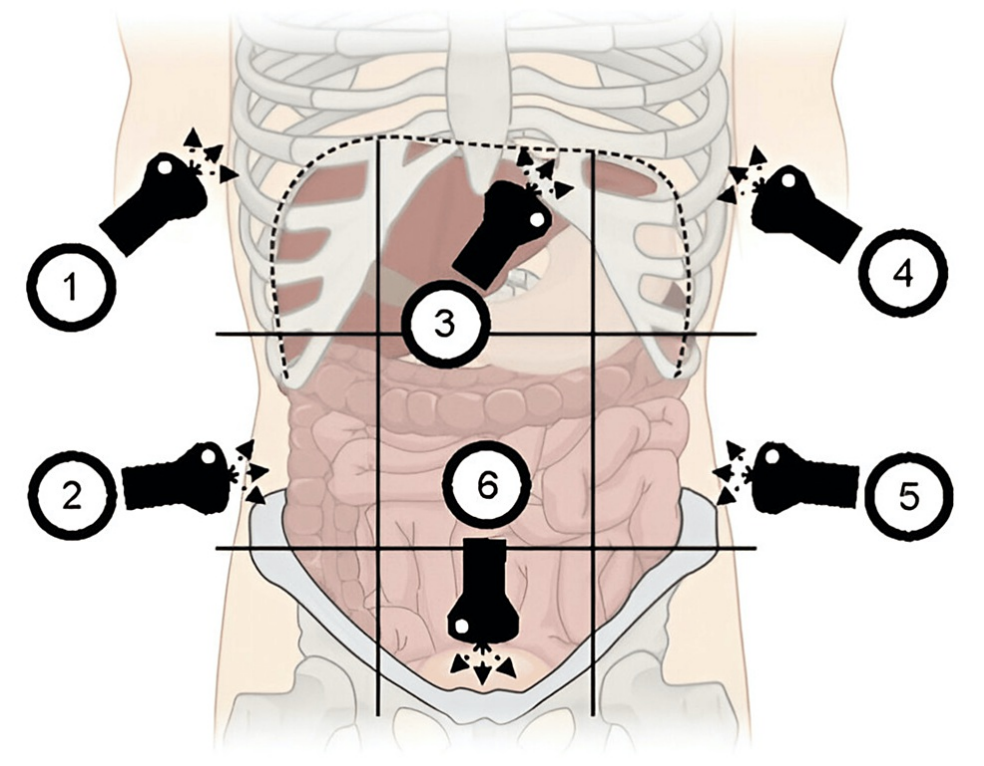
Protocol for focused assessment with sonography. Schematic image showing the protocol for focused assessment with sonography in cancer examination. Progression of scanning follows numbering from 1 to 6: 1. Right lung base and right upper quadrant abdomen. 2. Righ lower quadrant abdomen. 3. Subxifoid cardiac view. 4. Left lung base and left upper quadrant abdomen. 5. Left lower quadrant abdomen. 6. Suprabupic pelvic view. The original figure is presented in the article “The focused assessment with sonography in cancer examination.” Written permission to include the figure in this article was obtained from the original publisher (Dr. Benjamin T. Galen MD, Editor-In-Chief, Pocus Journal).

POCUS has demonstrated its utility in the context of palliative care across several particular applications. Research has indicated that POCUS enhances diagnostic accuracy and reduces the time required for diagnosing different illnesses. Furthermore, POCUS has become a standard of care for numerous procedures due to its ability to enhance patient safety. A proposed eight-point methodology for the placement positions of palliative medicine POCUS probes has been proposed (Figure 1) based on several applications that will be discussed [31].

POCUS pulmonary and cardiac evaluation in home-based palliative care

Differential Diagnosis of Dyspnea

Dyspnea is a prevalent primary symptom frequently reported by palliative patients, particularly during the preterminal stage of progressive illnesses. The aforementioned situation places a significant strain on individuals receiving medical care and their respective families, resulting in visits to emergency departments and hospitalizations, heightened levels of anxiety and apprehension, and a decline in overall well-being [32]. Acute dyspnea poses a significant challenge in promptly and precisely distinguishing its underlying causes due to its complex nature and the wide range of potential contributing factors [33].

Radiologic and laboratory findings are commonly utilized in the conventional management of dyspnea. Nevertheless, an increasing body of evidence has emerged, indicating the potential diagnostic accuracy of lung ultrasound employing handheld devices in the management of acute dyspnea. This technique has shown promise in distinguishing between several underlying causes of this condition [34].

A comprehensive assessment of the lungs can be conducted with an exploratory examination that involves evaluating six symmetrical locations on both sides of the chest, encompassing the anterior, lateral, and posterior surfaces [35].

A typical depiction of the lungs has a reverberation artifact characterized by hyperechogenic A-lines that run parallel to the pleura. When the A-line artifact is observed in conjunction with lung sliding, which refers to the observable motion of pleural plaques, it suggests the presence of a well-ventilated lung. During a thorough examination of the lungs, it is seen that pathological lesions in immediate proximity to the pleura are more effectively visualized compared to those located in the deeper layers as the air between the lesion and the chest wall prevents its visualization. The examination of the pleural interface involves the assessment of various alterations in the pleura, including subpleural consolidations or pleural thickening. Additionally, the evaluation encompasses the identification of pleural effusion, as well as the absence of the characteristic motion of the pleura, which may indicate the presence of pneumothorax [36].

There exists a substantial body of evidence indicating the utility of POCUS in the distinction of dyspnea. This value extends beyond the identification of pleural effusion or pneumothorax and encompasses conditions such as pneumonia, atelectasis, congestive heart failure, pulmonary embolism, and cardiac tamponade [37]. It is imperative to recognize that the execution of diagnostic pulmonary POCUS has specific challenges in individuals with pre-existing lung pathology [38]. In a newly published study on handheld POCUS, it was observed that palliative care patients experiencing dyspnea commonly had significant ascites, with pleural effusion or a mix of both being the more prevalent findings [26].

The inclusion of a dedicated protocol for evaluating palliative care across diverse clinical situations could be seen as an enhancement to the curriculum of POCUS in palliative care. This addition has the potential to promote increased uniformity in treatment choices within the field of palliative care [39].

While care often prioritizes symptom relief for patients, certain cases may necessitate medical interventions to achieve optimal control of symptoms. It is imperative to exercise judicious management to achieve clinically significant benefits while minimizing the generation of harm in particular patients. This consideration should be present throughout all stages of treatment, with the aim of avoiding futile interventions. The handheld devices’ portability enables attending palliative physicians to frequently and easily examine patients experiencing dyspnea during home visits. This examination method is characterized by its rapidity, repeatability, non-ionizing nature, and cost-effectiveness, with minimal discomfort to the patient [40]. Hence, POCUS plays a crucial role in facilitating timely clinical decision-making at the patient’s bedside, expediting the provision of relief for those experiencing dyspnea. Additionally, POCUS helps to avert unnecessary hospital transfers to emergency medical units to conduct diagnostic procedures that may not be warranted [41].

Pleural Effusion

Pleural effusions frequently contribute to dyspnea and discomfort among individuals receiving palliative care, particularly those diagnosed with lung and breast cancers. A post-mortem study has indicated that pleural effusions were present in around 15% of patients who succumbed to malignancies [42].

The diagnostic accuracy of clinical examination for detecting pleural effusions is directly proportional to the size of the effusion. It is exceedingly unlikely to diagnose a pleural effusion when the volume of fluid is less than 300 mL. The diagnostic performance of anteroposterior projection in standard chest radiography is limited, and it is only effective in cases where the volume of pleural effusion exceeds 200 mL [43].

In contrast, ultrasound exhibits a sensitivity of 100% in detecting effusions over 100 mL and is capable of detecting even smaller quantities as low as 20 mL, which is a very good performance when compared with computed tomography imaging [44].

Ultrasound imaging enables the evaluation of effusion characteristics, typically appearing as anechoic but occasionally exhibiting diverse echogenicity patterns, including the presence of septa and loculations in cases of malignant effusions. This technique additionally enables the assessment of the lung parenchyma, specifically for the presence of atelectasis, condensations, or masses, as well as the thoracic wall, specifically for the presence of masses, lytic costal lesions, nodularity, or pleural thickening [45]. Video 1 presents a pleural effusion on ultrasound.

**Video 1:** Pleural effusion. These thoracic ultrasound images were acquired with a handheld device in the coronal plane and demonstrate a supradiaphragmatic homogeneously anechoic effusion without internal echoes or septations. It is also possible to see that the space above the hemidiaphragm does not mirror the echogenicity of the liver. A triangular hyperechoic image is compatible with compressive pulmonary atelectasis and is seen “swimming” in the sizable pleural effusion, depicting the “jellyfish sign.” Patient consent was obtained for the use of anonymous medical data in this paper.

Moreover, employing this methodology enables the estimation of pleural effusion volume. Pleural effusion tends to collect in the dependent regions of the thorax, particularly in the posterolateral costophrenic recess, when the patient assumes a sat or semi-sitting position. The assessment of pleural effusion can be conducted using either qualitative or quantitative methods. Among the various quantification techniques available, the Balik formula is widely utilized. This formula involves obtaining a transverse view at the posterior axillary line and measuring the maximum distance between the parietal and visceral pleura in millimeters. Subsequently, this measurement is multiplied by 20 to estimate the volume of pleural effusion in milliliters [46].

Finally, the utilization of ultrasound allows healthcare professionals to assess the necessity of invasive interventions to address pleural effusions, hence mitigating patients’ symptom load and enhancing their overall quality of life [47].

Pneumonia

Pneumonia is a significant problem within the context of palliative care patients. The identification of this condition poses challenges due to atypical clinical presentation. Furthermore, it is associated with a decline in the individual’s quality of life as a result of the prevalence of distressing symptoms, notably dyspnea, and the frequent requirement for hospitalization [48].

While chest radiography remains the primary diagnostic modality, its sensitivity is reported to be 65% and its specificity is 81%. In contrast, bedside lung ultrasound is a more suitable modality for the diagnosis of pneumonia, with a sensitivity of 96% and specificity of 85%, particularly when the lesions are situated in the peripheral lung regions [49].

Multiple ultrasound findings indicate a potential diagnosis of pneumonia. These include the identification of a consolidation that exhibits echogenicity resembling that of the liver, commonly referred to as “hepatization” of the lung. Another finding, known as the “shred sign,” involves the previously smooth border of the lung becoming jagged, irregular, and blurred. Additionally, the presence of a hyperechogenic air bronchogram within the consolidation or a focal B-line pattern can also suggest pneumonia [50]. It is imperative to appropriately interpret these findings in conjunction with the clinical presentation [51]. Video 2 illustrates an ultrasound of pneumonia.

**Video 2:** Pneumonia. This thoracic ultrasound shows the absence of normal pulmonary parenchyma A-line artifacts; instead, it is possible to see the consolidated lung as a structure with a similar sonographic appearance to the liver (“lung hepatization”) with internal arborizing and hyperechoic linear structures consistent with air bronchogram. This appearance is suggestive of bacterial lobar pneumonia. A moderate pleural effusion is also seen. Patient consent was obtained for the use of anonymous medical data in this paper.

The utilization of handheld devices enables palliative care physicians to promptly diagnose patients in their homes, thereby circumventing avoidable hospital transfers and futile procedures. This expedites the implementation of appropriate treatment, such as antibiotic therapy or supportive measures aimed at enhancing symptom management. It is important to note that these therapeutic decisions are tailored to each patient, prioritizing their comfort and overall quality of life [52].

Congestive Heart Failure

Heart failure is a medical disorder characterized by its chronic and progressive nature, incurability, and, ultimately, its lethality. This condition is characterized by elevated rates of morbidity and mortality, which escalate with each instance of hospitalization, comparable to, if not surpassing, numerous forms of cancer [53]. Patients diagnosed with terminal heart failure commonly experience elevated rates of hospital readmission. This can be attributed to a decline in cardiac output or the accumulation of fluid, both of which are linked to a substantial symptom burden and a gradual deterioration in their overall quality of life [54].

POCUS handheld devices have the potential to assist palliative care clinicians in detecting extravascular lung fluid at the patient’s bedside. This is particularly relevant as extravascular lung fluid is known to contribute to the appearance of B-line abnormalities in ultrasound imaging. The observed artifacts have a vertical orientation and possess a hyperechogenic quality, such as laser beams. They originate from the pleural line and extend downward to the bottom boundary of the screen. The presence of three or more of these lines may indicate the presence of pathological conditions [55]. There is a strong correlation between the quantity of B-line artifacts and both the clinical status of the patient and the severity of heart failure according to the New York Heart Association classification [56]. Video 3 displays B-lines on ultrasonography.

**Video 3:** B-lines. This thoracic ultrasound was performed by a handheld device and shows several examples of B-lines. These lines arise from the pleural line and are well-defined hyperechoic comet-tail artifacts that extend indefinitely and erase A-lines, not disappearing with increasing depth, and moving in concordance with the lung. Patient consent was obtained for the use of anonymous medical data in this paper.

It is crucial to recognize that B-lines should not be considered exclusive indicators of cardiological interstitial edema. Instead, their association with heart failure decompensation should only be made within the appropriate clinical context and in conjunction with cardiac pathologic ultrasound findings, such as reduced overall contractility and an accompanying enlargement of heart cavities. Video 4 illustrates an ultrasound of a patient with severe heart failure.

**Video 4:** Heart failure. These cardiac ultrasound images (apical four-chamber view) were acquired with a handheld device and present a global contractility decrease of the left ventricle (LV). Normal findings would entail a global inward systolic excursion of the LV wall toward a central point, accompanied by wall thickening. In this case, the LV is hypodynamic as there is impaired wall contraction/inward systolic excursion, which may help infer that LV function is significantly impaired. Patient consent was obtained for the use of anonymous medical data in this paper.

The integration of ultrasound observations with clinical evaluation appears to yield more precision compared to the present diagnosis methodology reliant on chest radiography and cardiac biomarkers [57].

Using this approach, palliative care physicians can recognize the underlying cause of dyspnea in patients inside the comfort of their own homes. They can differentiate this cause from other potential factors, such as the worsening of chronic obstructive lung disease. Furthermore, they can rapidly optimize medical treatment by administering appropriate interventions, such as parenteral loop diuretics, vasodilators, or opioids. Hence, by its contribution to the reduction of symptom load, it effectively prevents hospital readmissions and unnecessary invasive procedures, while enhancing the quality of life for patients. This enables them to be treated in their preferred place of care and death [58].

Pericardial Effusion and Cardiac Tamponade

Palliative care patients may exhibit many causes of pericardial effusion. It can be related to pericardial metastases that arise from neoplasms originating in the lung, breast, esophagus, and lymphoma, or it can be associated with drug toxicity resulting from chemotherapy, immunotherapy, and radiotherapy [59].

The clinical evaluation, specifically the classic Beck triad, consisting of arterial hypotension, jugular venous distension, and muffled cardiac tones, exhibits limited sensitivity in diagnosing cardiac tamponade. Furthermore, while the presence of a paradoxical pulse is a more sensitive clinical indicator, it lacks specificity. Similarly, the diagnostic accuracy of electrocardiogram or chest X-ray findings in predicting the presence of tamponade is suboptimal [60]. In contrast, ultrasonography exhibits a sensitivity and specificity of 90% in the identification of effusion, even when performed by inexperienced physicians [61].

While it is typically recommended to conduct a comprehensive examination in all planes during a regular echocardiogram, the approach of choice in the context of palliative care is the subcostal or subxiphoid approach. The presence of pericardial effusion is characterized by the identification of an anechoic structure with varying distribution surrounding the heart chambers. Neoplastic involvement can result in changes in echogenicity, with the potential to observe the presence of septa and a pericardium that is thicker or exhibits nodular characteristics. When the diameter between the parietal and visceral leaflets at the conclusion of the diastole is over 2 cm, it is classified as severe, indicating a fluid volume beyond 700 mL [62].

The term “swinging heart” is used to describe the phenomenon of cardiac oscillation that occurs in cases of significant pericardial effusion and tamponade. The phenomenon typically involves the contraction of the right ventricles during the relaxation phase of the cardiac cycle, resulting in compromised hemodynamics. Additionally, it is characterized by an enlarged inferior vena cava measuring more than 20 mL, which exhibits no changes in size during respiratory cycles. To diagnose tamponade, it is important to correlate ultrasonography results with the existence of arterial hypotension, tachycardia, and indications of reduced cardiac output [63]. Video 5 presents a pericardial effusion on ultrasound.

**Video 5:** Pericardial effusion. This cardiac ultrasound (subcostal view) was performed by a handheld device. It shows a large pericardial effusion with systolic right atrial and diastolic right ventricular collapse. There was also an abnormal respiratory variation of transmitral and transtricuspid flows (not shown) and a distended inferior vena cava (not shown). These signs are compatible with a cardiac tamponade. Patient consent was obtained for the use of anonymous medical data in this paper.

With this approach, it is possible to reduce the delay in performing a pericardiocentesis in cases when it is deemed clinically necessary.

POCUS gastrointestinal evaluation in home-based palliative care

Ascites

The occurrence of ascites in patients receiving palliative care can be attributed to several factors and is often complex. It may be a manifestation of hypervolemia in cases of advanced chronic heart or renal failure when the patient’s response to diuretic treatment is no longer effective. It can be malignant in oncological patients due to peritoneal carcinomatosis and in that case usually resistant to diuretic treatment. Alternatively, it can arise owing to albumin deficit or portal hypertension attributed to significant liver metastases or portal vein obstruction and then be treatable with diuretics [64]. Video 6 illustrates an ultrasound of hepatic metastases.

**Video 6:** Hepatic metastases. This abdominal ultrasound shows several rounded, well-defined, slightly hypoechoic lesions with a hyperechoic halo, and mass effect (distortion of the hepatic vein). These were hepatic metastases from a primary lung adenocarcinoma. Patient consent was obtained for the use of anonymous medical data in this paper.

The symptoms commonly linked to malignant ascites can cause severe impairment, leading to a notable decline in quality of life. Palliative care physicians face difficulties in effectively managing the symptoms of ascites. The accumulation of fluid in the peritoneal cavity can result in painful abdominal distension, anorexia, nausea and vomiting, dyspnea, fatigue, peripheral edema, and excessive weight gain. These symptoms can lead to reduced mobility in patients and the escalation of analgesics, although sometimes only suboptimal relief of discomfort is achieved with those measures [65].

The process of clinical examination entails the assessment of conventional indicators, such as dullness and flank distention. However, its effectiveness in detecting ascites is limited, presenting a sensitivity of 75% and a specificity of 57%. Furthermore, the detection of these findings necessitates a minimum fluid volume of 1,500 mL, which can be aggravated when it involves obese patients or substantial meteorism [66].

Hence, ultrasonography is considered the preferred diagnostic modality for confirming the existence of ascites and estimating their volume. The observed collection is typically homogenous and anechoic. However, in cases of malignant ascites, its echogenicity may be modified, exhibiting characteristics such as septa, loculations, peritoneal and mesenteric implants, and potentially enhanced vascularization of the parietal peritoneum [67]. Video 7 displays an ascites on ultrasonography.

**Video 7:** Ascites. This abdominal ultrasound shows a large volume of intraperitoneal anechoic fluid. There is no floating debris suggestive of exudative, hemorrhagic, or neoplastic ascites. It also does not present septations characteristic of an inflammatory or neoplastic etiology. The bowel loops and liver are seen posteriorly. Patient consent was obtained for the use of anonymous medical data in this paper.

The presence of ascites carries both diagnostic and prognostic consequences. To make informed therapeutic decisions, it may be required to perform ultrasound-guided ascites aspiration and evaluate biochemical indicators of peritoneal carcinomatosis together with cytological examination [68].

Bowel Obstruction

Acute or subacute bowel obstruction is a relatively frequent condition observed in individuals with advanced gastrointestinal tract or pelvic malignancies, particularly in cases when unresectable peritoneal carcinomatosis is present [69].

Bowel obstruction may present as constipation, vomiting, abdominal pain or distension, or peritoneal signs that point toward the diagnosis and can lead to substantial morbidity and mortality [70].

While abdominal plain films are commonly employed as an initial diagnostic tool for bowel obstruction, the diagnostic accuracy of POCUS performed by the attending clinicians is found to be favorable when compared to traditional radiography studies. POCUS demonstrates a sensitivity of 90% and a specificity of 97% when used for the detection of small bowel obstruction [71].

Common sonographic observations often consist of the presence of dilated and fluid-filled small bowel loops, the absence of peristalsis, the distinctive “to and fro” peristalsis motion that characterizes the pendular movement of intestinal contents, or a collapsed colonic lumen [72].

With this approach, POCUS has the capability to deliver prompt and comprehensive clinical information directly at the patient’s bedside, avoiding excessive and redundant imaging procedures and eliminating the need for patient relocation and contrast administration [73]. Moreover, it can aid in the prompt administration of targeted symptomatic treatment [74]. It can also be beneficial in determining the necessity for further cross-sectional imaging in patients without a documented history of intra-abdominal malignancy. The demonstration of obstruction is of prognostic significance, as evidenced by a six-month survival rate of roughly 50% in cases where the obstruction is operable compared to a mere 8% in cases where the obstruction is deemed inoperable [75]. Hence, POCUS needs to be regarded as the most suitable initial diagnostic tool within the context of palliative care.

POCUS renal and vesical evaluation in home-based palliative care

Hydronephrosis and Acute Urinary Retention

Urologic emergencies, such as upper and lower urinary tract obstruction, are commonly observed in patients receiving palliative care. These emergencies carry significant implications, including the induction of distressing symptoms such as lower abdominal and flank pain, increased susceptibility to infections, and the potential development of progressive renal failure in cases of bilateral obstruction. Furthermore, these emergencies hold notable prognostic significance in various malignancies [76].

POCUS has demonstrated significant utility in palliative care for assessing patients presenting with diminished or absent urinary output and/or acute kidney injury (AKI). By employing POCUS, clinicians can effectively, quickly, and non-invasively discern whether the AKI is attributable to post-renal causes, including urinary tract obstruction [77].

The assessment of urinary tract obstruction includes the examination of both kidneys and the bladder, employing two imaging planes, namely, transverse and longitudinal.

When evaluating the kidneys, the primary focus is on determining the presence of hydronephrosis. Hydronephrosis is a condition characterized by the enlargement of the renal pelvis and calyces as a result of a blockage in the flow of urine. This obstruction can be caused by intrinsic factors, such as lithiasis or urothelial tumors, and extrinsic factors, such as lymphadenopathy or tumors located in the pelvic or retroperitoneal regions. During the early phases of obstruction, the renal pelvis appears anechoic. If the obstruction progresses, the calyces dilate and, in severe and persistent obstruction, the pelvis and calyces appear anechoic, the medullary pyramids and cortex experience thinning, and the pyelocaliceal architecture is no longer preserved [78]. Video 8 illustrates an ultrasound of hydronephrosis.

**Video 8:** Hydronephrosis. This abdominal ultrasound was performed with a handheld device and shows moderate dilatation of the renal pelvis (>10 mm) and calyces that appear blunted/convex. There are some areas of slight cortical/parenchymatous thinning. This is a case of a moderate hydronephrosis. Patient consent was obtained for the use of anonymous medical data in this paper.

According to available reports, physicians have demonstrated the potential to attain proficiency in evaluating hydronephrosis, exhibiting diagnostic capabilities comparable to those of radiologists, given the necessary training [79].

Despite its limitations in determining the exact cause of upper urinary tract obstruction, POCUS enables clinicians to quickly detect hydronephrosis with a high level of accuracy sensitivity and specificity approaching 90%. This facilitates timely multidisciplinary discussions regarding the necessity for additional advanced imaging or interventions, such as palliative percutaneous nephrostomy or suprapubic cystostomy. It is preferable to perform these procedures under the guidance of ultrasound [80]. While these procedures have the potential to enhance pain management and effectively address symptoms associated with renal failure, it is crucial to consider the potential risks, patient expectations, and prognosis to make an informed decision about their implementation [81].

When assessing the bladder, it is imperative for clinicians to conduct the calculation of intravesical urine volume as it enables the definitive diagnosis of acute urinary retention [82].

Bladder outlet obstruction leading to urinary retention can occur due to various mechanical factors affecting the bladder neck (such as a pelvic tumor or clot), prostate (such as prostatic hypertrophy), or neuronal damage of the bladder (such as spinal cord injury). Additionally, urinary retention can also be attributed to the adverse effects of certain medications, specifically anticholinergic drugs, opioids, and antidepressants [83].

POCUS assessment holds significant clinical implications in palliative care, as it facilitates prompt interventions, such as the placement of a urinary catheter for therapeutic purposes, leading to the expected alleviation of symptoms. Additionally, it has the potential to prevent unnecessary catheterization in individuals experiencing non-obstructive oliguria. This is significant as catheterization can lead to discomfort and traumatic injury to the urethra and serve as a potential source of infection. Video 9 illustrates a bladder outlet obstruction.

**Video 9:** Bladder outlet obstruction. This pelvic ultrasound was performed with a handheld device and reveals a significantly distended bladder. It is possible to visualize hyperechoic endoluminal content in a dependent position that may be related to pyuria or hematuria. These features may indicate the need for the placement of a urinary catheter. Patient consent was obtained for the use of anonymous medical data in this paper.

POCUS can also be employed for evaluating the positioning and functionality of urinary catheters in patients who exhibit reduced urine production. In the event that the catheter remains properly positioned and there exists just a minimal amount of urine surrounding the balloon of the catheter, it can be inferred that the reduced urine production is not attributable to any obstruction or malfunction of the catheter. Consequently, alternative factors should be investigated to identify the underlying cause [84]. Video 10 presents a bladder ultrasound after the placement of a urinary catheter.

**Video 10:** Urinary catheter. This abdominal ultrasound was performed with a handheld device showing a properly positioned urinary catheter with the tip and inflated balloon visible within the bladder lumen. Patient consent was obtained for the use of anonymous medical data in this paper.

According to reports, a concise training program has demonstrated the potential for palliative care physicians and nurses to effectively utilize handheld ultrasound devices for managing the placement of transurethral catheters and monitoring post-catheter removal residual urine volumes [85].

The utilization of handheld devices during home visits enables palliative care clinicians to promptly make bedside determinations regarding the insertion or repositioning of urinary catheters. This intervention effectively reduces the duration of symptomatic discomfort and diminishes the necessity for emergency room visits in patients with clinically suspected lower urinary tract obstruction [28].

POCUS vascular evaluation in home-based palliative care

Deep Vein Thrombosis and Venous Thromboembolism

Venous thromboembolism (VTE) is a frequent complication in individuals receiving palliative care, encompassing both cancer patients, as malignancy serves as an autonomous risk factor [86], and non-cancer patients, due to the presence of comorbidities and limited mobility [87].

The adverse prognostic implications of deep vein thrombosis (DVT), specifically the significant mortality rate observed in untreated individuals as a result of the progression to pulmonary thromboembolism (PTE), along with the accompanying morbidity associated with treatment, particularly the risk of bleeding, underscore the importance of promptly identifying or ruling out this condition [88].

The preferred method for diagnosing DVT in the lower limb is focused compression ultrasonography. This technique is often performed at the common femoral vein and popliteal vein levels, where compression is used to occlude the normal hypoechogenic venous lumen [89].

The diagnosis of DVT is established when there is no full collapse of the veins or when echogenic material (thrombus) is present within the veins [90]. The technique of extended compression ultrasound involves the extension of the ultrasound scanning sequence above the inguinal ligament and down to the confluence of the calf vein. The presence of color Doppler can be used to confirm the absence of flow inside the veins. However, it should be noted that certain ultrasound handheld devices may not have Doppler flow capabilities, and the lack of this feature does not hinder the diagnostic process [91]. Video 11 illustrates an ultrasound of a DVT.

**Video 11:** Deep vein thrombosis. This ultrasound shows a non-compressible femoral vein with hyperechoic intraluminal content compatible with deep vein thrombosis. It is also possible to visualize thrombus extension to the great saphenous vein (the smaller vein originating from the femoral vein). Patient consent was obtained for the use of anonymous medical data in this paper.

According to available reports, the outcomes of venous decompression ultrasound conducted by a skilled practitioner exhibit similarity to those of Doppler ultrasonography administered by a radiologist. The examination has a steep learning curve, resulting in a sensitivity rate of 100% and a specificity rate of 91%-98% [92]. In addition, ultrasonography is widely regarded as a secure and dependable method for diagnosing DVT, as there is no substantiated data suggesting that it elevates the likelihood of triggering a thromboembolic event [93].

The DVT diagnosis can be the starting point for diagnosing PTE. Consequently, it is crucial to conduct lung ultrasound and focused cardiac ultrasound to identify right ventricular dysfunction, which serves as an indirect indicator and aids in risk assessment for determining appropriate therapeutic approaches. In the context of acute massive embolism, it is important to look for specific cardiac findings, such as the dilation of the right ventricle lumen, its predominance over the left ventricle, and the abnormal displacement of the interventricular septum toward the left ventricle [94]. Video 12 illustrates an ultrasound compatible with PTE.

**Video 12:** Pulmonary thromboembolism. This cardiac ultrasound (short-axis view) shows a severely dilatated and hypokinetic right ventricle, flattening of the interventricular septum, and systolic collapse of the left ventricle. It is possible to visualize the “D sign,” which is an ultrasound finding that shows the left ventricle as a D-shaped structure as a result of the increased right ventricular pressure causing an interventricular septum displacement toward the left side of the heart. These findings are compatible with pulmonary embolism and are associated with a bad prognosis as they indicate that the patient may have a massive pulmonary embolism with significant right ventricular strain. Patient consent was obtained for the use of anonymous medical data in this paper.

Using handheld devices, POCUS can be performed directly at the patient’s bedside, thereby obviating the need for an unwarranted hospital transfer. This approach effectively minimizes the psychological burden experienced by both patients and their caregivers [95]. The identification of a positive result indicating the presence of VTE allows further imaging testing to be streamlined, targeted, or potentially avoided. This allows physicians to promptly make treatment decisions directly at palliative care facilities [96]. In the setting of palliative care, the clinical significance of VTE is contingent upon its association with a patient-reported symptom burden. This consideration must be weighed alongside factors such as life expectancy, performance status, bleeding risk, and patient motivation to arrive at an appropriate treatment decision [97].

POCUS-guided interventions in home-based palliative care

Ultrasound-Guided Thoracocentesis

Ultrasound guidance has been found to be superior to the conventional approach in cases where thoracentesis is deemed appropriate. This method enables the identification of the optimal site for catheter placement, which is particularly crucial due to the common occurrence of non-free malignant pleural effusions [98].

In cases where the fluid chamber is of substantial size, the thoracocentesis operation can be conducted in a static real-time manner. This entails the operator determining the distance between the skin and the effusion, and thereafter marking the puncture site without directly visualizing the needle throughout the procedure. However, it is advisable to carry out the technique for small, septated, or loculated pleural effusions with the use of dynamic real-time ultrasonography control, in which the operator continuously monitors the needle’s position during the procedure [99]. Video 13 presents a complex pleural effusion on ultrasound.

**Video 13:** Complex pleural effusion. This thoracic ultrasound demonstrates a supradiaphragmatic heterogenous effusion. It is possible to visualize the plankton sign, which refers to internal echoes with slow and whirling motion. Septations are also present and should rule out a transudative effusion in favor of an exudative process, namely, infection, hemothorax, or chylothorax. Patient consent was obtained for the use of anonymous medical data in this paper.

**Figure 2:**
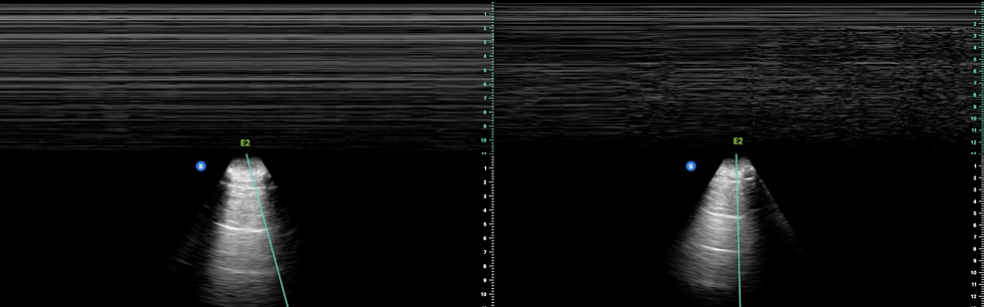
Pneumothorax. These are thoracic M-mode ultrasound images (upper part of the images), accompanied inferiorly by the respective B-mode selected location. On the left, there is only a barcode/stratosphere sign (M-mode evidence of pneumothorax) indicating no lung sliding due to air interposition. On the right, it is possible to see transitions from the seashore sign (M-mode evidence of normal lung sliding) to the barcode/stratosphere sign. This allows the determination of the “lung point sign,” an ultrasound finding that represents the transition between sliding lung and pneumothorax. This sign is highly specific for pneumothorax, showing the point where the visceral pleura begins to separate from the parietal pleural at the margin of the pneumothorax. According to its location, the “lung point” may also indicate the size of the pneumothorax. Patient consent was obtained for the use of anonymous medical data in this paper.

After the completion of the procedure, it is possible to perform an additional ultrasound scan to assess the pleural line sliding and rule out the presence of an iatrogenic pneumothorax. The identification of this complication can be accomplished through the use of ultrasound examination, which exhibits a notable degree of specificity and sensitivity. This condition is distinguished by several key observations, including the existence of the A-line artifact, the absence of pleural lung sliding, the presence of the stratosphere sign in the M-mode option, and the identification of the lung point [100]. Figure 2 illustrates an ultrasound image of a pneumothorax.

With this approach, ultrasound aids in diminishing the occurrence of post-interventional complications, specifically pneumothorax and hemothorax, to a level below 1%. Additionally, it effectively decreases the incidence of “dry taps” in pleural effusions that obliterate less than half of the hemidiaphragm [101].

The growing accessibility of portable devices enables the safe implementation of this procedure on an outpatient basis [102], particularly in the residences of patients receiving palliative care. This approach offers immediate alleviation of symptoms [103] and eliminates the necessity of transferring patients to the radiology department. Consequently, there is no longer a need for pre-procedural marking of the designated area or post-procedural chest radiography to rule out pneumothorax [104]. Ultrasound has the potential to assist in the identification of individuals with malignant pleural effusion who are likely to benefit from pleural intervention, as well as those who may have a limited response to such treatment [105].

In situations involving recurrent pleural effusion necessitating repeated aspiration, the utilization of ultrasound-guided pigtail catheter insertion may be a viable option. This approach is comparable in efficacy to the conventional method of chest tube insertion and offers the advantage of being performed within a community setting, obviating the need for patient transfer to a hospital setting [106]. Nevertheless, it is imperative to carefully evaluate the potential advantages and disadvantages of this invasive procedure on a case-by-case basis for every patient [107]. The strong advice provided by the current recommendations to utilize thoracic ultrasonography guidance for all pleural procedures is justified by the various advantages it offers.

Ultrasound-Guided Paracentesis

The use of paracentesis in the context of palliative care involves the alleviation of physical symptoms by decompressing a tense ascites-filled abdomen. This procedure can effectively mitigate distressing symptoms and can be employed as a supplementary therapeutic approach, obviating the need for the escalation of opioids. The technique should be carried out with a deliberate aim to enhance comfort and the overall quality of life [108].

The use of ultrasound guidance during medical procedures instills a higher level of assurance in the practitioner’s ability to perform the process, while concurrently diminishing the occurrence of complications resulting from the procedure from 4.7% to 1.4% [109]. In contrast, ultrasound imaging provides a definitive means of identifying the presence of fluid, preventing the need for potentially hazardous procedures in cases when the presence of fluid is not significant and paracentesis would yield little therapeutic benefit. This contributes to a safer and equally imperative decision-making process within the context of palliative care [110]. Video 14 presents complex ascites on ultrasound, in which paracentesis would be of little utility and could result in severe iatrogenic complications.

**Video 14:** Complex ascites. This abdominal ultrasound shows a large volume of intraperitoneal anechoic fluid. Several internal septations indicate a possible inflammatory or neoplastic etiology. Given that there is evidence of loculated ascites, it may be neither safe nor beneficial to perform a paracentesis. Patient consent was obtained for the use of anonymous medical data in this paper.

With this approach and using handheld devices, the execution of ultrasound-guided paracentesis in non-hospital settings, particularly within patients’ residences, is both viable and secure. This approach eliminates the need for patient transfer and admission in cases of symptomatic ascites, while also preventing unnecessary transfers when paracentesis is not warranted. Consequently, this method engenders satisfaction among patients and their families [24,111].

As the etiology of ascites is frequently untreatable, it is reasonable to anticipate the recurrence of ascites and the necessity for periodic paracentesis in patients receiving palliative treatment. Hence, ultrasound imaging can be employed to facilitate the placement of indwelling tunneled catheters in such specific scenarios. Nevertheless, it is crucial to evaluate the risk-to-benefit ratio in specific therapeutic contexts to make decisions that prioritize the best interest of the patient [112].

Ultrasound-Guided Venous Access

The occurrence of periprocedural pain during vascular access is commonly documented in medical literature. It is worth noting that this discomfort is frequently not adequately addressed, resulting in suboptimal pain management. Moreover, repeated efforts at vascular access can potentially induce a heightened sensitivity to pain, known as hyperalgesia [113]. In a palliative care setting, the administration of various intravenous medications and the potential veno-toxic effects associated with some treatments, such as chemotherapy, might introduce additional complexities.

The use of ultrasound guidance for venous access has been shown to enhance the probability of successful cannulation in individuals with difficult peripheral venous access [114]. This approach is associated with reduced levels of stress and pain during the placement procedure [115], as well as a decreased occurrence of complications in hospice and home care environments.

The approach recommended is to use a linear high-frequency probe for ultrasound-guided entry, employing either the in-plane or out-of-plane strategy, in cases where two or more previous attempts have been made or when a patient is identified to have difficult peripheral access [116].

A recent review has provided evidence that non-physician medical personnel, such as paramedics and nurses, can attain proficiency in the placement of peripheral venous access using ultrasound guidance with relatively little training. This finding suggests that this technique is readily accessible and has the potential for widespread use [117].

Ultrasound-Guided Analgesic Strategies

The use of interventional procedures for pain management serves as a beneficial supplement to conventional analgesic medication, as opiate analgesia may not be able to provide consistent and sustainable long-term analgesia for individuals experiencing chronic pain without causing undesirable adverse effects such as constipation, nausea, or somnolence [118].

The use of bedside ultrasound has the potential to facilitate the implementation of pain management strategies in outpatient settings, enabling effective pain control.

The utilization of isolated ultrasound-guided peripheral nerve blocks has been documented as an effective method for providing sufficient palliative analgesia across individual dermatomes. This approach has demonstrated the ability to decrease the occurrence of unsuccessful blocks and associated complications, serving as a valuable technique for enhancing accuracy, safety, and patient comfort [119].

This approach holds significant importance in the management of cancer-related pain. The utilization of a bedside ultrasound-guided technique for celiac plexus neurolysis has the potential to effectively manage intra-abdominal pain in a specific subset of patients receiving palliative care, particularly individuals with advanced upper abdominal cancers. It can lead to enhanced pain management, decreased reliance on narcotic medications, and an overall improvement in their quality of life [120].

The application of ultrasound guidance in the procedure of superior hypogastric plexus neurolysis has proven to be a valuable tool in effectively managing pelvic cancer pain in patients with advanced gynecologic malignancies [121]. The use of ultrasound-guided tissue-plane blocks and plexus blocks has demonstrated encouraging outcomes in the management of neuropathic or metastatic bone pain that is insufficiently managed with oral analgesia [122].

It is worth noting that musculoskeletal disease is a prominent contributor to pain and functional limitations among the palliative care population, often surpassing their underlying terminal diagnosis in terms of impact [123]. The use of ultrasound is gaining significance in the fields of arthrocentesis and corticosteroid and local anesthetic infiltrations. This approach offers enhanced success rates, reduced procedural discomfort, and effective symptomatic relief while avoiding the potential complications associated with systemic steroid therapy [124]. In fact, adequate treatment of musculoskeletal pain at the end of life is a valuable prospect for enhancing the overall quality of life experienced by these patients [25].

Limitations of point-of-care ultrasound in home-based palliative care

The application of POCUS in a palliative care setting does have a few limitations that warrant consideration.

The use of ultrasound in medical examinations depends on the skills and experience of the operator. There exists a degree of variability in the way different operators perform and interpret POCUS findings. This variability is influenced by various factors, including specific patient characteristics, but mostly hinges on the expertise of the operator. The presence of variability in examinations can have an impact on the sensitivity and specificity of each individual examination. It is important to take this variability into account when interpreting POCUS findings to avoid errors in clinical management [125].

This constraint can be surmounted through comprehensive training under the guidance of experts in frequent and highly cost-effective situations. POCUS can indeed be effectively integrated into the training curriculum of palliative care physicians, mirroring the approach that has been suggested for the curriculum of internal medicine residency programs. To build a successful POCUS curriculum, residency programs need multiple resources, including faculty expertise, didactic materials, equipment, and hands-on experience. A comprehensive POCUS program may be most efficacious when it involves an array of pedagogical approaches, including online and in-person didactic lectures, pre- and/or post-course quizzes, simulations utilizing comprehensive ultrasound models and procedure-oriented mannequins, training in live healthy models, and direct scanning of actual patients. Developing a personal portfolio comprising a compilation of archived examinations enables instructors to retrospectively evaluate and provide feedback on the images [126]. According to research, the confidence of residents in their ability to use ultrasound during invasive procedures was found to increase as a result of ultrasound training, improving procedural skills, and potentially enhancing patient safety. There were also improvements in image acquisition and interpretation, as well as a more adequate integration of these findings into clinical decision-making, determining POCUS-driven changes in clinical management [127]. It is important to note that POCUS training is currently thriving within the community of undergraduate medical education, preparing medical school graduates to more easily use POCUS in their future careers. The addition of standardized POCUS instruction into palliative care training programs is a crucial next step to enable its use in this setting, being a potential catalyst for further education, research, and quality assurance [128].

An alternative approach to supporting less experienced examiners is the use of ultrasound teleconsulting, a method that should be pursued on a case-by-case basis in individualized patients [129]. Nowadays, it is technically feasible with handheld devices to remotely transmit ultrasound images and a visual impression of both patient and probe positioning to an expert. This enables real-time POCUS examination optimization and enhances its quality [130]. While there are some worldwide courses on palliative POCUS, it is important to note that sustaining the acquired skills requires ongoing local mentorship, appropriate archiving of images and reports, and effective supervision to ensure the quality of diagnostic outcomes [131].

In addition to the deficiency in faculty expertise, a significant obstacle lies in the limited availability of equipment and adequate infrastructure within the specialized palliative care environment, with a mere fraction of units possessing such resources [14]. The widespread adoption of ultrasound technology in healthcare settings in the future is expected to have a positive impact on the affordability of equipment and the accessibility of portable devices for point-of-care applications. In fact, POCUS is a cost-effective and potentially portable imaging modality that holds significant promise for applications in resource-constrained settings where alternative imaging modalities might be limited. The only absolute requirements for its application are a trained user, an electrical power source, a portable ultrasound machine, and an ultrasound gel. Nevertheless, in these healthcare settings, the prohibitive cost and limited availability of commercially available ultrasound gel may restrict the quantity and quality of scans that clinicians can perform, despite the presence of necessary equipment. To address this issue, research has been undertaken to develop cost-effective alternatives for commercially available ultrasound gel using locally sourced materials. These alternatives have demonstrated comparable image adequacy, quality, detail, and resolution to commercial gels [132].

Concerning the feasibility and utility of POCUS in out-of-hospital settings, several studies have been conducted demonstrating the feasibility of deploying portable ultrasound in remote, austere environments. Given the demanding nature of medical care in remote or underdeveloped locations, it is important to increase clinical diagnostic capacity and prevent potentially unnecessary evacuations to higher-level facilities solely for imaging studies. Ultrasound may enable prompt and targeted treatment to be initiated at the location. It can also facilitate the differentiation of patient needs and the selection of the most appropriate evacuation hospital [133]. The feasibility of POCUS in primary care practice has also been investigated, and a recent study showed positive results mainly attributable to its short acquisition time that is compatible with routine visits and a wide range of applicable clinical scenarios. Furthermore, this study demonstrated the efficacy and safety of POCUS, as evidenced not only by the elevated accuracy of initial diagnoses that remained consistent with definite diagnoses achieved subsequently but also by the low occurrence of false negatives [134]. When considering home ultrasound, feasibility research is scarce. However, a case-control study has established that conducting ultrasound examinations in the residences of vulnerable patients is not only feasible but also reduces diagnostic uncertainty and delay in care in a statistically significant manner when compared with hospital care. The majority of patients solely required clinical follow-up by their physician, and among those patients who required supplementary tests or referrals, the definitive diagnosis consistently mirrored the outcomes of the ultrasound performed at home [135]. While the diverse range of healthcare settings in which POCUS has been implemented may restrict the generalizability of results, this underscores its versatile nature and the necessity to further investigate and expand its potential applications. The current body of literature examining the use of POCUS in palliative care primarily comprises case reports and case series. Focused prospective research to examine POCUS as a first diagnostic strategy in this particular population is scarce [26,30]. Hence, it is imperative to conduct research that specifically examines the evaluation of patient-centered outcomes, such as peri-interventional pain, the necessity for patient relocation, readmission rates, or symptom amelioration, particularly in the context of utilizing POCUS within acute palliative care settings [136].

## Conclusions

POCUS represents an expansion of the clinical examination, constituting its fifth pillar, and should be acknowledged as a complementary tool to enhance the efficacy of thorough history-taking and physical examination. The primary purpose of this tool is to answer dichotomous questions in situations of high clinical profitability, specifically within the realm of direct patient care. Hence, the objective of POCUS is not intended to supplant clinical judgment or a regulated and structured radiological assessment.

Ultrasound devices have become compact, portable, and easily accessible, facilitating the provision of palliative care services in home-based outreach settings. Handheld devices are highly suitable for addressing the special requirements in the provision of care for palliative patients due to their ability to enhance physicians’ decision-making capabilities, minimize diagnostic delays, offer prognostic and evolutionary insights, and mitigate complications associated with specific palliative interventions.

Its performance by a patient-known and trusted professional who is already aware of the surrounding clinical context contributes to meeting patients’ desire to spend their final stage of life in their home environment. This approach helps prevent futile invasive procedures, unnecessary transfers to crowded hospital emergency departments, and excessive financial expenditures on hospital healthcare.

Hence, it plays a significant role in reducing the burden on palliative care patients, facilitating prompt and efficient alleviation of symptoms, and ultimately enhancing the patient’s well-being and quality of life.

However, the decision to use imaging in inpatient palliative care necessitates a pragmatic evaluation of the advantages of potentially enhanced clinical knowledge against futility and the potential for harm. Some of the limitations of homecare ultrasound are the lack of confidence and training of the clinicians and the restricted availability together with associated expenses of equipment. Therefore, it is imperative to incorporate this contemporary and valuable tool into the realm of palliative care practice, thereby promoting widespread, structured, easily accessible, high-quality, skill-oriented, and accredited training.

It is time to let POCUS become not only the first diagnostic modality but also the cornerstone of bedside evaluation and part of the standard of care in the management of palliative care patients.
